# Reasoning-Driven Food Energy Estimation via Multimodal Large Language Models †

**DOI:** 10.3390/nu17071128

**Published:** 2025-03-24

**Authors:** Hikaru Tanabe, Keiji Yanai

**Affiliations:** Department of Informatics, The University of Electro-Communications, 1-5-1 Chofugaoka, Chofu 182-8585, Tokyo, Japan; tanabe-h@mm.inf.uec.ac.jp

**Keywords:** image-based food energy estimation, multimodal large language models, volume injection, daily food intake tracking

## Abstract

**Background/Objectives:** Image-based food energy estimation is essential for user-friendly food tracking applications, enabling individuals to monitor their dietary intake through smartphones or AR devices. However, existing deep learning approaches struggle to recognize a wide variety of food items, due to the labor-intensive nature of data annotation. Multimodal Large Language Models (MLLMs) possess extensive knowledge and human-like reasoning abilities, making them a promising approach for image-based food energy estimation. Nevertheless, their ability to accurately estimate food energy is hindered by limitations in recognizing food size, a critical factor in energy content assessment. **Methods:** To address this challenge, we propose two approaches: fine-tuning, and volume-aware reasoning with fine-grained estimation prompting. **Results:** Experimental results on the Nutrition5k dataset demonstrated the effectiveness of these approaches in improving estimation accuracy. We also validated the effectiveness of adapting LoRA to enhance food energy estimation performance. **Conclusions:** These findings highlight the potential of MLLMs for image-based dietary assessment and emphasize the importance of integrating volume-awareness into food energy estimation models.

## 1. Introduction

Monitoring daily food intake is a critical step towards achieving health-related goals such as dieting and bodybuilding. Accurate food energy estimation is particularly important for supporting these efforts, yet traditional methods like Food Diaries, 24 h Dietary Recalls (24HR), and Food Frequency Questionnaires (FFQ) rely on participants to self-report their intake, making the data prone to various errors and biases [1,2]. These self-reporting approaches suffer from limitations such as recall bias, misreporting due to social desirability, and the burden of detailed tracking, often leading to inaccurate energy intake estimations. Furthermore, the requirement for extensive manual input discourages long-term adherence, limiting their effectiveness in real-world dietary monitoring.

This challenge highlights the need for more efficient and user-friendly solutions that reduce the user burden, while maintaining high accuracy. With the increasing availability of smartphones and AR-enabled devices, capturing food images has become a practical alternative for dietary monitoring. By leveraging computer vision methods, image-based food energy estimation automates dietary tracking, minimizing user effort and reducing reliance on memory-based reporting [3,4,5,6,7]. This approach offers an intuitive and less intrusive means of monitoring food intake, making it particularly advantageous for individuals who struggle with traditional self-reporting methods.

However, despite its potential, image-based food energy estimation remains challenging due to the diversity in food types and portion sizes, which significantly impact energy estimation accuracy. Current methods often fail to adapt to such variations effectively [5].

Multimodal Large Language Models (MLLMs) offer a promising solution to these challenges. MLLMs combine visual and textual reasoning capabilities, enabling them to identify diverse food items and contextualize visual information for tasks like food energy estimation. Recent advancements have demonstrated their potential in solving food-related tasks by leveraging extensive knowledge base and reasoning capabilities [8,9]. However, existing MLLMs struggle with accurately recognizing food volume, which is crucial for determining energy content [10].

In this study, we propose an approach that builds upon MLLMs to improve image-based food energy estimation. By introducing a fine-tuning strategy and volume injection with fine-grained estimation prompting, our approach addresses the limitations of current methods in handling food diversity and volume estimation. Experimental evaluation on the Nutrition5k dataset [6] demonstrates the effectiveness of these strategies in enhancing the quality of energy estimation.

We review the methodology from our previous works [11,12] and incorporate a fine-grained prompting strategy to improve food energy estimation. Additionally, we conduct several ablation studies, including an evaluation of the effectiveness of adapting LoRA for food energy estimation performance.

The main contributions of this study are summarized as follows:

- We introduce an approach that leverages fine-tuning and volume injection to enhance the reasoning and recognition capabilities of MLLMs for image-based food energy estimation.
- We propose a fine-grained estimation prompting method to address the challenges of food volume recognition in MLLMs.
- We evaluated the proposed approach on the Nutrition5k dataset, showing significant improvements over baseline methods and discussing its strengths and limitations.

## 2. Related Work

### 2.1. Image-Based Food Energy Estimation

Estimating food energy content from images has been widely studied, due to its potential applications in health and nutrition [7]. Two primary approaches exist for this task: size-based methods, and direct estimation methods.

Size-based methods typically involve multiple steps, starting with segmenting food regions in an image, estimating the food category, and then calculating the volume or mass of the food regions. The energy content is derived from these intermediate estimations. This approach enables precise consideration of food quantity, a critical factor for food energy estimation accuracy.

Determining the actual size of food in images has been approached through various techniques. One common strategy involves using everyday objects as reference points, such as credit cards or wallets [3], chopsticks [13], or even rice grains [14]. Augmented reality (AR) has also been leveraged, where virtual anchors enable users to estimate food dimensions interactively [4]. Beyond these object-based methods, advancements in depth estimation have further refined food size and volume calculations. DepthCalorieCam [5] improved caloric estimation by integrating depth cameras with segmentation models, while implicit surface reconstruction techniques enabled the creation of detailed 3D food meshes, capturing both the food and dish with high fidelity [15].

Despite their potential, size-based methods are often limited in their ability to handle diverse food types. For instance, DepthCalorieCam is restricted to estimating the caloric content of only three food categories [5], significantly limiting its applicability.

Direct estimation methods bypass intermediate steps and use deep learning models trained end-to-end to directly estimate energy content. For example, Ege et al. [16] applied a multi-task learning framework based on VGG16 [17] to simultaneously estimate food category, ingredients, cooking methods, and energy content. While this approach simplifies the pipeline, it struggles to account for variations in food quantity, leading to potential inaccuracies when portions differ. Furthermore, these methods require extensive labeled datasets for training, imposing significant annotation costs.

In this study, we address the limitations of both approaches by leveraging Multimodal Large Language Models (MLLMs) and integrating volume estimation capabilities. Our method combines a promptable segmentation model, an open-set segmentation model, and monocular depth estimation to achieve high-quality zero-shot food energy estimation, reducing the reliance on annotated training data.

### 2.2. Multimodal Large Language Models (MLLMs)

Recent advancements in Large Language Models (LLMs) have demonstrated their ability to achieve remarkable performance across a wide range of language tasks by scaling model parameters, data, and computational resources [18]. These models exhibit emergent capabilities, where performance improves significantly at certain scaling thresholds [19]. Extending these models to handle visual information has led to the development of Multimodal Large Language Models (MLLMs), which integrate vision and language for enhanced reasoning.

Several MLLMs have been developed with superior performance across vision–language tasks. Flamingo [20] integrates visual and textual features using gated cross-attention, enabling it to handle diverse image and video tasks. LLaVA [8] employs linear layers to map visual features into a format compatible with LLMs and applies visual instruction tuning for task-specific improvements. Models like BLIP-2 [21], MiniGPT-4 [22], and InstructBLIP [23] further refine this approach, incorporating specialized modules such as Q-Former and leveraging instruction-following data for robust generalization.

In the food domain, FoodLMM [9] has demonstrated strong performance across various tasks, including food energy estimation. However, its accuracy is limited by insufficient volume recognition capabilities. In this study, we build on the reasoning and generalization capabilities of MLLMs and introduce enhancements to improve their ability to estimate caloric content from food images by incorporating explicit volume estimation and fine-tuning strategies.

## 3. Methods

In this study, we implement two approaches that enhance the food energy estimation capability of MLLMs: fine-tuning (Section 3.1), and volume injection with fine-grained prompting (Section 3.2).

### 3.1. Fine-Tuning MLLMs

**Figure 1:**
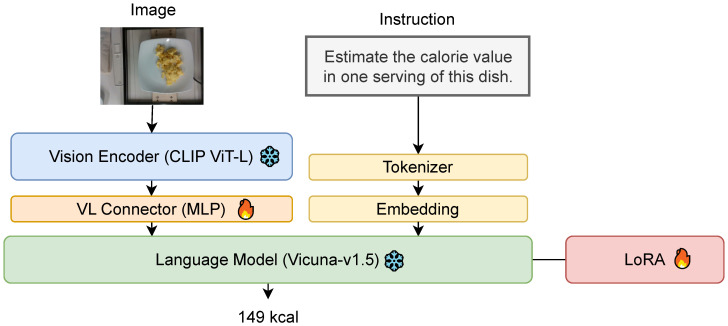
The architecture of MLLM.

We train MLLMs based on LLaVA-1.5 [24], to adapt their reasoning capabilities for image-based food energy estimation. The MLLM architecture consists of three main components (Figure 1):

Visual Encoder: The input image is encoded into visual features using the OpenAI CLIP ViT-L encoder [25]. Vision–Language Connector: A two-layer MLP transforms the visual features to match the dimensions of text token embeddings. Language Model: The transformed visual features and text token embeddings are processed by Vicuna-v1.5 [26], a fine-tuned LLM, which outputs the estimated energy value.

To train the MLLMs, we used the Nutrition5k dataset [6], which includes paired food images and energy annotations. The dataset includes 3265 annotated food images with energy information. The dataset was converted into instruction-following format, where the instruction prompts the model to estimate energy values, and the response provides the energy value in a structured format (e.g., [[300]] calories). This consistent format facilitates extraction of energy content using regular expressions. Fine-tuning is performed using LoRA [27] for efficient adaptation with reduced computational overhead.

### 3.2. Volume Injection

To overcome the limitation of MLLMs in recognizing food volume, we apply a volume injection approach. This involves a dedicated volume estimation module and the integration of volume information into the MLLM.

#### 3.2.1. Overall Architecture

**Figure 2:**
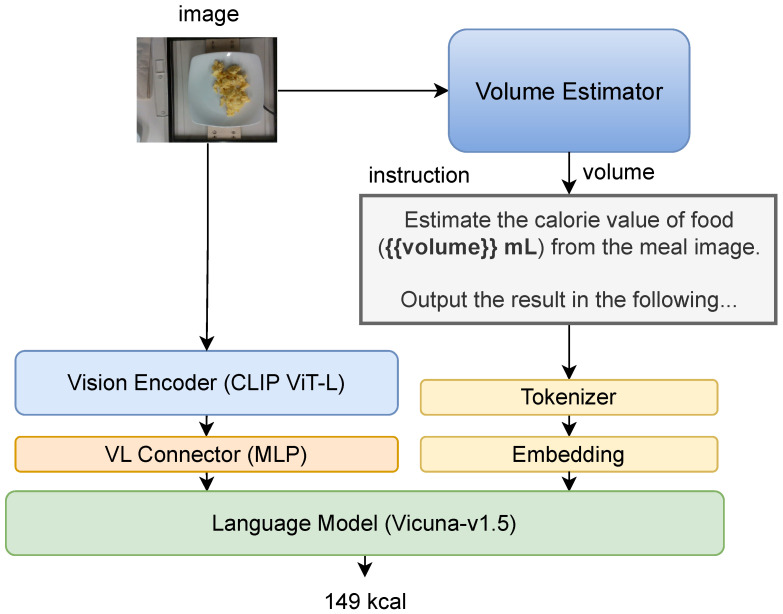
Overall architecture of volume injection approach (based on LLaVA-1.5 [24]).

**Figure 3:**
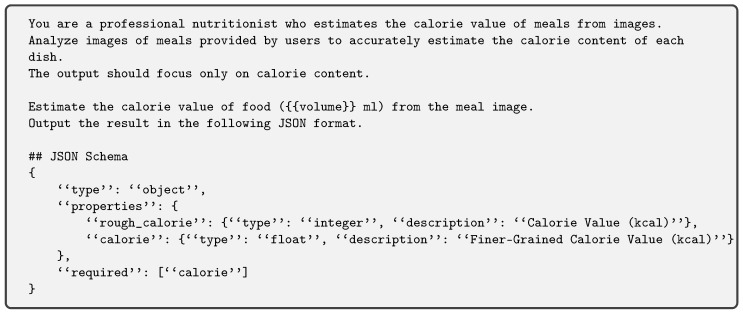
Instruction prompt with fine-grained estimation prompting.

The integration of the volume estimation module with the MLLM is shown in Figure 2. The estimated food volume is injected into the MLLM via instruction. Specifically, the instruction includes a placeholder {{volume}}, which is replaced with the calculated volume value. This approach allows the MLLM to reason food energy content with explicit consideration of food volume, improving its estimation accuracy. Figure 3 is the instruction prompt used for GPT-4V and 4o. To self-refine the accuracy of the estimated energy value, the value is asked in two-step format. The estimated energy value is output as JSON format, defined in the JSON Schema. For LLaVA-1.5, we remove the specification of the JSON format and put the following sentence:

Return single calorie value in the following format: "[[x]] calories.’’

due to a lack of capability to follow the JSON format.

#### 3.2.2. Volume Estimation Module

**Figure 4:**
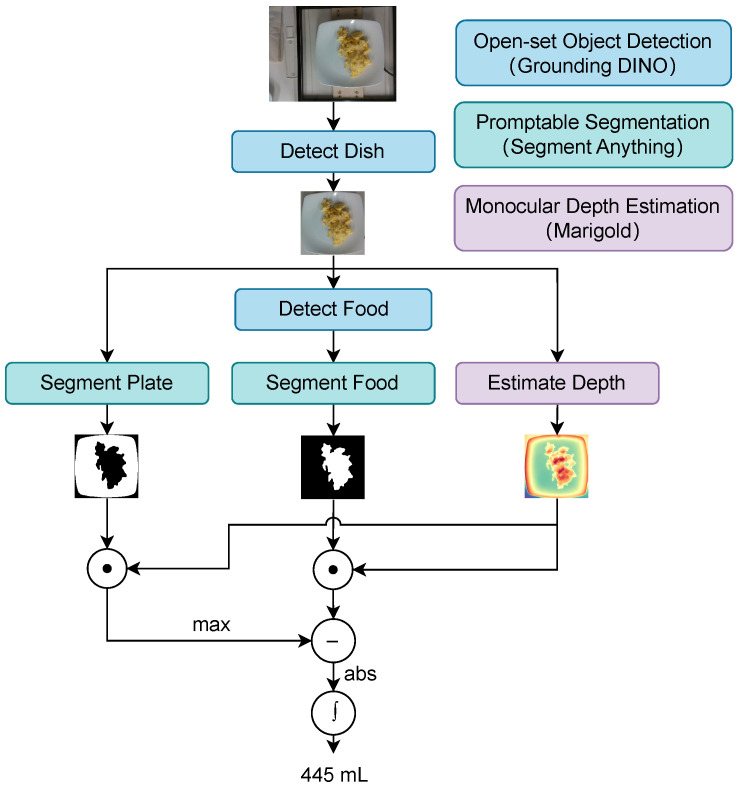
Architecture of the volume estimation module. This module processes a food image to estimate the food volume, which is then injected into the MLLM.

The volume estimation module processes food images to estimate the volume through the following steps (Figure 4):

First, the dish’s bounding box is detected using Grounding DINO [28], an open-set object detection model. Then, the Segment Anything Model (SAM) [29] generates masks for the food and the plate within the bounding box. A depth map is also estimated using Marigold [30], a monocular depth-estimation model.

The actual volume of the food is calculated using the extracted masks and depth map. The depth values corresponding to the food region are isolated by taking the Hadamard product of the depth map and the food region mask. The height of the food above the dish’s reference plane is computed by subtracting the depth of the dish from the depth values of the food region. The per-pixel volume is calculated using the actual height and area of each pixel, and the total volume is obtained by summing the contributions of all pixels, as shown in Equation (1):

(1) $$V=\sum_{i=1}^{n}\sum_{j=1}^{m}D_{ij}A_{ij}$$

here, $D_{ij}$ and $A_{ij}$ represent the height and area of each pixel at row *i* and column *j*. *n* and *m* indicate the height and width of the image, respectively.

## 4. Experimental Results

We conducted experiments to evaluate the proposed approaches using the Nutrition5k dataset [6], which is designed for nutritional analysis of food images. It comprises 3265 top-down food images, each paired with detailed nutritional information, including food energy content. For this study, we used 2759 samples for the training split, with the remaining 506 samples for the test split.

The evaluations included supervised food energy estimation, and zero-shot food energy estimation with volume injection.

### 4.1. Experiment Settings

We fine-tuned LLaVA-1.5-7B and 13B models for supervised setting using the training split of Nutrition5k, referring to the trained models as LLaVA-1.5-7B FT and 13B FT. The training was conducted using the AdamW optimizer with linear warmup and cosine decay, a peak learning rate of $2\times 10^{-4}$, and a batch size of 64.

The models were evaluated on the test split of Nutrition5k. For text generation, the temperature value was set to 0 to ensure deterministic outputs. Failed extractions were retried with a temperature of 0.2 up to five times.

The volume injection approach was evaluated by combining the proposed food volume estimator with LLaVA-13B, GPT-4V, and 4o. Since these models were not fine-tuned on Nutrition5k, this evaluation focused on zero-shot performance. Similar extraction rules were applied in failed cases as in the fine-tuning evaluation.

### 4.2. Fine-Tuning Results

**Figure 5:**
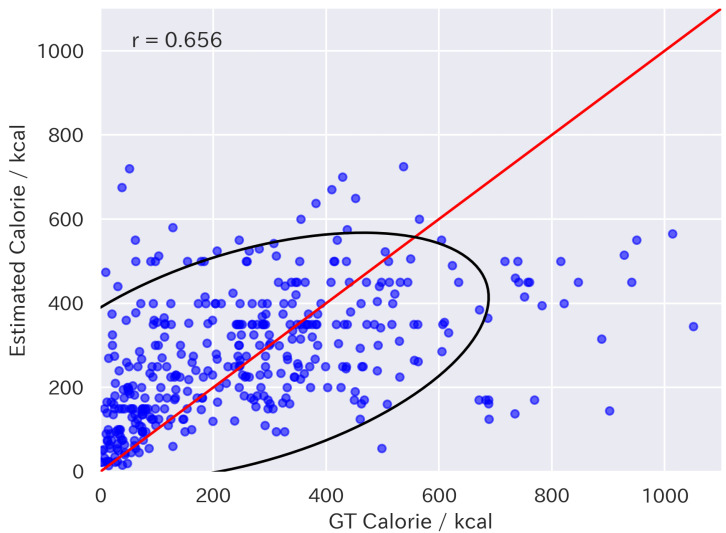
Scatter plot of energy values estimated by LLaVA-1.5-13B. The red line indicates equality between estimated and ground-truth values. The black line represents the 95% confidence ellipse.

**Figure 6:**
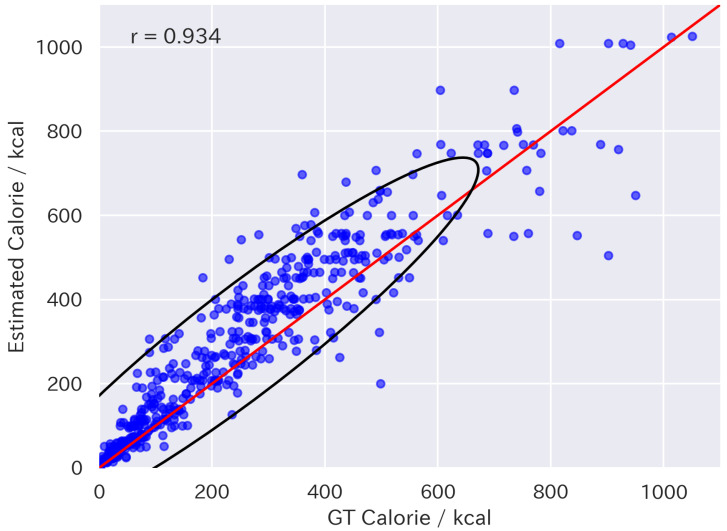
Scatter plot of energy values estimated by LLaVA-1.5-13B FT. The red line indicates equality between estimated and ground-truth values. The black line represents the 95% confidence ellipse.

Table 1 presents the results of fine-tuning. The results were evaluated using Mean Absolute Error (MAE), Mean Absolute Percentage Error (MAPE), and the correlation coefficient between the estimated values and the ground truth. The direction of the arrows indicates that a smaller value represents better performance for MAE and MAPE, while a larger value indicates better performance for the correlation coefficient. The LLaVA-1.5 FT models outperformed the baseline models, including GPT-4V and 4o for all metrics. In particular, LLaVA-1.5-13B FT also outperformed supervised expert models such as Google-nutrition-monocular [6] and FoodLMM [9], in terms of MAE. Figure 5 and Figure 6 show the distributions of the energy values estimated by LLaVA-1.5-13B and 13B FT, respectively. They indicate that the fine-tuning approach was effective for improving food energy estimation.

Table 2 compares the food energy estimation results under different training strategies. The model trained with LoRA outperformed the model using full-parameter tuning across all metrics, demonstrating its effectiveness for improving accuracy, while reducing training costs. Since LoRA requires fewer trainable parameters than full-parameter tuning, it helps mitigate overfitting, especially when using a relatively small dataset such as Nutrition5k.

### 4.3. Zero-Shot Results with Volume Injection

**Figure 7:**
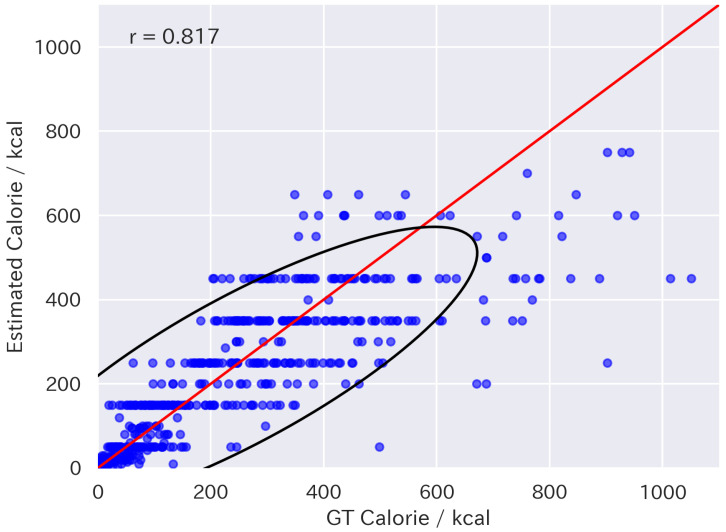
Scatter plot of energy values estimated by GPT-4o. The red line indicates equality between estimated and ground-truth values. The black line represents the 95% confidence ellipse.

**Figure 8:**
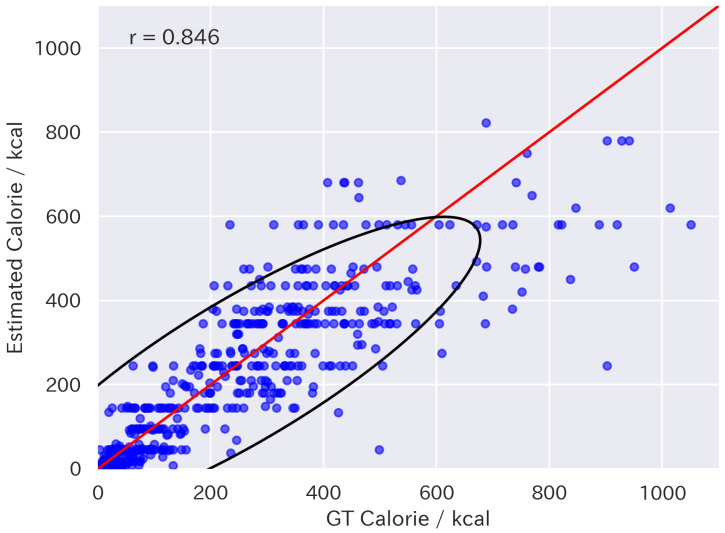
Scatter plot of energy values estimated by GPT-4o with volume injection. The red line indicates equality between estimated and ground-truth values. The black line represents the 95% confidence ellipse.

Table 3 shows the results of the zero-shot food energy estimation. In particular, combining the proposed volume injection with GPT-4o led to improvements in all metrics. Figure 7 and Figure 8 are the distributions of estimated energy values by GPT-4o and 4o with volume injection, respectively. They illustrate that volume injection with fine-grained estimation prompting improved the variance in estimated energy values, which resulted in more accurate estimation.

**Figure 9:**
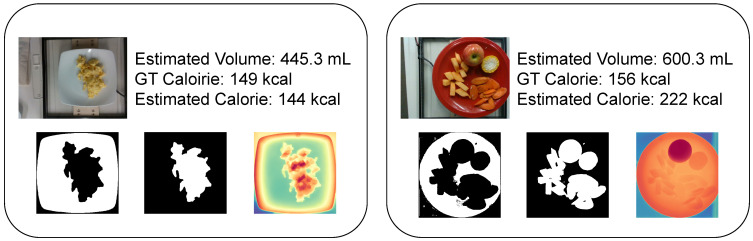
Outputs of the volume estimation module: object detection, segmentation, and depth estimation (the estimated energy values are the outputs by GPT-4V with volume injection).

Additionally, Figure 9 demonstrates the segmentation and depth estimation outputs, highlighting the volume estimation module’s ability to localize food regions and compute volumes effectively. For object detection and segmentation, both the dish and food regions were accurately extracted, indicating high-quality estimation. In depth estimation, variations in uneven surfaces within the image were well captured. Additionally, in images containing multiple food items, areas with differing heights exhibited distinct depth values compared to their surroundings. These results highlight the effectiveness of the volume estimation module in localizing food regions and accurately computing their volumes.

## 5. Discussion

### 5.1. Challenges in Volume Estimation

**Figure 10:**
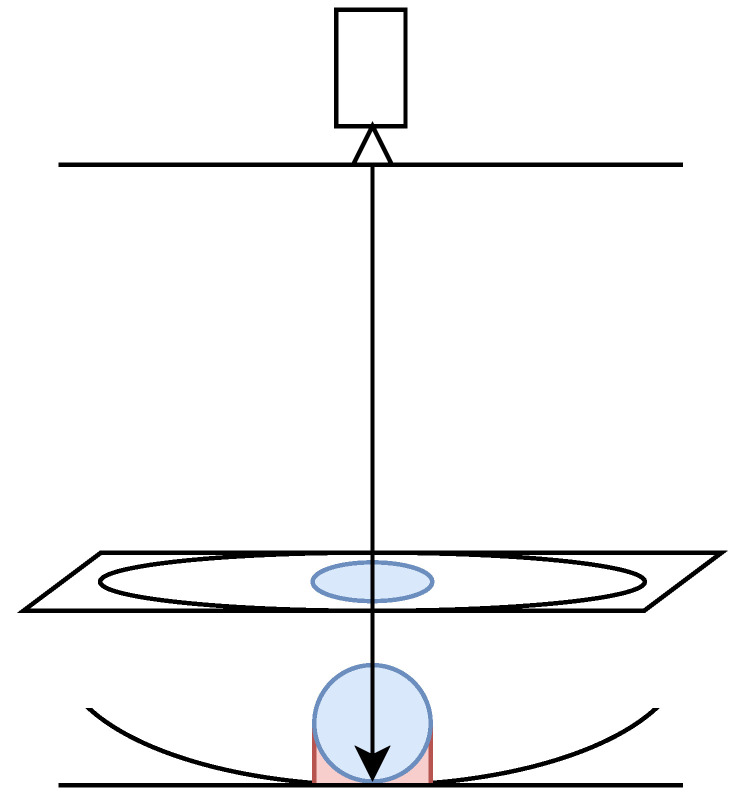
Overestimation of volume by the food volume estimator when the food is assumed to be spherical. Blue: food region, Red: excess region.

The food volume estimation module had challenges in overestimation of food volume (Figure 10):

- The estimated volume between the bottom of the food and the reference plane of the dish could be over-calculated.
- If the lowest part of the dish is obscured by food, an incorrect reference plane for the dish may be selected.

To mitigate these issues, methods from prior research can serve as valuable references. For example, DepthCalorieCam [5] used a food mass regression model to adjust for overestimated volumes, resulting in more accurate food energy estimation. Similarly, Naritomi et al. [15] reconstructed high-quality 3D shapes of food and dishes to exclude the area beneath the food and the reference plane, ensuring accurate volume estimation.

While these approaches are promising, they require extensive food data for training. Adjusting overestimated volumes demands large-scale annotated volume data, while improving 3D reconstruction methods necessitates high-quality 3D shape data, which remains a challenge in the food domain.

In particular, recent advancements in 3D reconstruction, such as Neural Radiance Fields (NeRF) [31] and 3D Gaussian Splatting [32], offer promising directions for addressing the limitations of volume estimation. These methods can generate detailed 3D representations of objects from 2D images, potentially overcoming the limitations of current 3D reconstruction models in the food domain. Integrating these techniques into the volume estimation process is expected to improve the accuracy of volume measurements.

### 5.2. Improving Commonsense Reasoning in MLLMs

Another potential enhancement involves leveraging the commonsense knowledge of MLLMs to revise and correct unreasonable energy estimations. For example, under zero-shot conditions, the language model could be prompted to reevaluate its reasoning process when it produces energy values that deviate significantly from expected norms. By incorporating such prompts, MLLMs could refine their estimations and provide outputs that align better with real-world expectations.

### 5.3. Future Directions

In future work, integrating advanced 3D reconstruction techniques and fine-tuning MLLMs to better capture spatial information will be crucial. Additionally, exploring ways to reduce the reliance on large-scale annotated datasets could lower the barrier to implementing these improvements. By addressing these challenges, the proposed framework could achieve more accurate and robust food energy estimation.

Beyond technical improvements, expanding the application of the proposed framework to interdisciplinary healthcare settings presents a promising direction. In hospital environments, this technology could assist clinicians in estimating caloric intake, particularly in cases of hospital malnutrition, where precise dietary monitoring is critical [33]. Integrating this model into multidisciplinary healthcare-technology projects, such as those involving nutritionists, nurses, and engineers, could facilitate real-time dietary assessment and personalized nutritional interventions [34,35].

Moreover, establishing standardized protocols for data collection and model evaluation across different clinical settings will be essential to ensure the reliability and generalizability of AI-driven dietary assessment systems. Interdisciplinary collaboration will also be key in defining best practices for integrating these technologies into existing hospital workflows, minimizing disruption, while maximizing clinical utility. Furthermore, collaboration with nursing and engineering researchers could enable the development of integrated healthcare-technology systems that leverage food intake monitoring for patient management [36,37]. Applications in other medical-technological domains, such as metabolic disorder management and postoperative dietary tracking, also warrant exploration [38,39]. By extending the reach of this framework into healthcare and beyond, the impact of AI-driven dietary assessment can be maximized across various fields.

### 5.4. Discussion Summary

Despite the advancements achieved in this study, several limitations remain. First, the accuracy of volume estimation was affected by overestimation issues, due to the challenges in determining the reference plane of the dish and occlusions in food images. Future work could address these issues by incorporating improved 3D reconstruction techniques or refining volume estimation models with additional data. Second, while our approach enhances reasoning capabilities for food energy estimation, commonsense reasoning in MLLMs remains an open challenge, particularly in cases where the estimated values deviate significantly from expected norms. Incorporating self-refinement mechanisms or domain-specific constraints could further improve estimation accuracy. Lastly, our evaluation primarily relied on the Nutrition5k dataset, which, although diverse, may not fully represent real-world variations in food presentation and composition. Expanding evaluation to broader datasets and real-world scenarios will be necessary to ensure versatility. Addressing these limitations will be crucial for enhancing the robustness and applicability of MLLM-based food energy estimation models.

## 6. Conclusions

In this study, we applied MLLMs to food energy estimation, enhancing their capabilities by introducing fine-tuning and volume injection with fine-grained prompting. Through fine-tuning MLLMs and incorporating a food volume estimation module, our approach demonstrated improvements in food energy estimation accuracy. Evaluations on the Nutrition5k dataset showed that the proposed method outperformed baseline models and contemporary MLLMs, both in fine-tuned and zero-shot settings.

Despite these advancements, several challenges remain. The volume estimation module occasionally overestimated the food volume, due to inaccuracies in determining the reference plane of the dish or the height of the food. To address these limitations, future work could incorporate mass regression models and advanced 3D reconstruction techniques. These methods have the potential to refine the volume estimation process and improve model accuracy.

Additionally, more comprehensive evaluations of energy and volume estimation across a wider variety of food items are needed. Constructing a dataset that includes diverse food items annotated with volume and energy values would be a valuable contribution toward this goal.

## Figures and Tables

**Table 1:** Results of food energy estimation on Nutrition5k (fine-tuning).

Method	MAE/kcal ↓	MAPE/% ↓	r ↑
Google-nutrition-monocular [6]	70.6	26.1	-
LLaVA-1.5-7B	178.8	129.5	0.637
LLaVA-1.5-13B	177.1	92.8	0.656
GPT-4V	80.7	55.7	0.833
GPT-4o	82.7	46.7	0.817
FoodLMM FT [9]	67.3	26.6	-
LLaVA-1.5-7B FT	74.2	41.5	0.927
LLaVA-1.5-13B FT	64.3	39.8	0.934

**Table 2:** Comparison of full-parameter tuning and LoRA.

Method	MAE/kcal ↓	MAPE/% ↓	r ↑
LLaVA-1.5-13B FT (Full)	77.7	48.8	0.869
LLaVA-1.5-13B FT (LoRA)	64.3	39.8	0.934

**Table 3:** Results of zero-shot food energy estimation by MLLMs on Nutrition5k (volume injection).

Model	MAE/kcal ↓	MAPE/% ↓	r ↑
LLaVA-1.5-13B	109.6	92.8	0.656
GPT-4V	80.7	55.7	0.833
GPT-4o	82.7	46.7	0.817
LLaVA-1.5-13B w/vol	6122.7	6591.4	−0.041
GPT-4V w/vol	83.8	54.1	0.816
GPT-4o w/vol	78.8	43.4	0.846

## Data Availability

The dataset is available on the project page of Nutrition5k [6].
